# Channel-mediated astrocytic glutamate release via Bestrophin-1 targets synaptic NMDARs

**DOI:** 10.1186/1756-6606-6-4

**Published:** 2013-01-16

**Authors:** Kyung-Seok Han, Junsung Woo, Hyungju Park, Bong-June Yoon, Sukwoo Choi, C Justin Lee

**Affiliations:** 1 Center for Neural Science and WCI Center for Functional Connectomics, Korea Institute of Science and Technology (KIST), Seoul, Korea; 2 Neuroscience Program, University of Science and Technology (UST), Daejeon, Korea; 3 Division of Life science, School of Life Sciences and Biotechnology, Korea University, Anam-Dong, Seoul, Korea; 4 Department of Biological Science, Seoul National University, Seoul, Korea

**Keywords:** Astrocyte, Bestrophin-1, mEPSC, NMDAR

## Abstract

**Background:**

Astrocytes regulate neuronal excitability and synaptic activity by releasing gliotransmitters such as glutamate. Our recent study demonstrated that astrocytes release glutamate upon GPCR activation via Ca^2+^ activated anion channel, Bestrophin-1 (Best1). The target of Best1-mediated astrocytic glutamate has been shown to be the neuronal NMDA receptors (NMDAR). However, whether it targets synaptically or extra-synaptically localized NMDAR is not known.

**Findings:**

We recorded spontaneous miniature excitatory postsynaptic currents (mEPSCs) from CA1 pyramidal cells to test whether Best1-mediated astrocytic glutamate targets synaptic NMDAR. An agonist of protease activated receptor 1 (PAR1) was used to induce astrocytic Ca^2+^ increase and glutamate release. Firstly, we found that activation of PAR1 and subsequent release of glutamate from astrocyte does not alone increase the frequency of mEPSCs. Secondly, we found that mEPSC rise time is variable depending on the different electrotonic distances from the somatic recording site to the synaptic region where each mEPSC occurs. Two subgroups of mEPSC from CA1 pyramidal neuron by rise time were selected and analyzed. One group is fast rising mEPSCs with a rise time of 1 ~ 5 ms, representing synaptic activities arising from proximal dendrites. The other group is slowly rising mEPSCs with a rise time of 5 ~ 10 ms, representing synaptic events arising from glutamate release at synapses located in the distal dendrites. We used cell-type specific Best1 gene silencing system by Cre-loxP cleavage to dissociate the effect of neuronal and astrocytic Best1. Astrocytic Best1-mediated glutamate release by PAR1 activation did not affect decay kinetics, frequency, and amplitude of fast rising mEPSC. In contrast, PAR1 activation resulted in an NMDA receptor component to be present on slowly rising mEPSC, but did not alter frequency or amplitude.

**Conclusions:**

Our results indicate that astrocytic glutamate via Best1 channel targets and activates synaptic NMDARs.

## Background

Astrocytes provide structural scaffolding and nutrients to neurons as well as a mechanism for removing released neurotransmitters [1]. Recently, several studies have shown that astrocytes can be activated by sensory stimulation [2] or several pathological conditions including brain ischemia or inflammation [1,3-6]. These stimuli evoke increases in intracellular Ca^2+^ in astrocytes, which in turn elicit the release of active substances termed gliotransmitters [7-9]. These released gliotransmitters are known to be involved in modulating neuronal synaptic plasticity [3,10], synaptic scaling [7-9], and even excitotoxicity [11]. It has been reported that astrocytic glutamate is released by multiple mechanisms. However, we have recently reported that when G_αq_-coupled receptors such as PAR1 are activated, astrocytes release glutamate via Ca^2+^ activated anion channel, Bestrophin1 (Best1) [12].

*Bestrophin* is the gene linked to Best's vitelliform macular dystrophy and has been shown to encode a functional Ca^2+^-activated anion channel (CAAC) in nonneuronal tissue and peripheral neurons [13]. This Bestrophin-1 channel (Best1) is directly activated by submicromolar intracellular Ca^2+^ concentration and has an anion selective pore with single channel activities [13-20]. Recently, we have demonstrated that *Best1* encodes the most of CAAC in astrocytes of the CA1 hippocampus [21], that Best1 channels show unique permeability to large anions and osmolytes such as GABA and glutamate [12,22], that Best1 channels are selectively expressed at the astrocytic microdomains adjacent to glutamatergic synapse by immunogold electron microscopy [12], and that glutamate released via Best1 channel activates neuronal NMDA receptors in hippocampal CA1 pyramidal neurons [12]. However, whether astrocytic glutamate via Best1 channel targets synaptically localized NMDAR is unknown.

Miniature excitatory postsynaptic currents (mEPSCs) provide an elegant way to examine the mechanism at synaptic level because individual mEPSC represents one quantal release and activation of receptors localized at one synapse. Under the resting condition with a holding potential, Vh = −60 mV, the mEPSC kinetics represents current mediated entirely by synaptically localized AMPA receptor activation (rise) and desensitization (decay). The decay of AMPA receptor mediated mEPSCs can be well fitted to a single exponential decay function. The presence of normal levels of Mg^2+^ (1.5 mM) in the ACSF provides robust voltage dependent channel block of any synaptic NMDA receptors. However, under certain circumstances mEPSCs can contain a component by activation of synaptic NMDA receptor. These circumstances include when Mg^2+^ block of NMDA receptors has been relieved by either low Mg^2+^ or depolarization of the membrane potential of the postsynaptic compartment. The NMDA receptor component usually appears under voltage clamp as a slow, noisy inward current that is superimposed on the decay phase of mEPSCs. Under this condition, the decay of these mixed mEPSCs cannot be fitted well with a single exponential function, but with a sum of two exponential decay functions [23], the second of which reflects the time course of synaptic NMDA receptors.

In the present study, we explored the possibility that Best1-mediated astrocytic glutamate targets synaptically localized NMDAR by recording mEPSCs. We also utilized the cell type specific gene silencing system to dissociate astrocytic Best1. We demonstrate that Best1-mediated astrocytic glutamate by PAR1 activation causes an appearance of NMDAR component in slowly rising mEPSC, indicating targeting and activation of synaptic NMDARs.

## Results

### Two subgroup of mEPSC from CA1 pyramidal neuron

**Figure 1:**
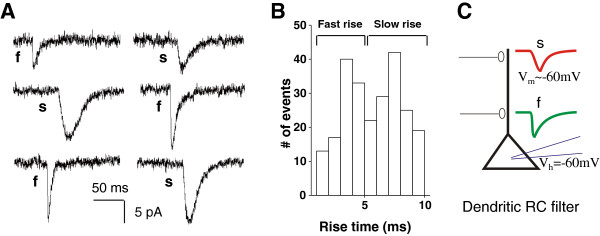
**Fast and slowly rising mEPSCs in CA1 pyramidal neuron. A**. Example traces of mEPSCs from three different slices show different rise time of mEPSCs recorded from CA1 pyramidal neuron. F is fast rising mEPSCs, and s is slowly rising mEPSCs. **B**. The histogram of rise times of 240 mEPSCs recorded from 3 cells for 8 minutes. Fast rise time of mEPSCs is 1 ~ 5 ms, and slow rise time of mEPSCs is 5 ~ 10 ms. **C**. Schematic diagram of fast rising and slowly rising mEPSCs. Somatic region of CA1 pyramidal neurons is whole cell patched under voltage clamp (holding potential −60 mV).

It has been well established that astrocytically released glutamate preferentially activates NMDAR in neighboring neurons in voltage- and Mg^2+^-dependent manner [24-28]. And recently we reported that astrocytically released glutamate via Best1 channel activates neuronal NMDARs in the hippocampal CA1 pyramidal neurons [12]. To examine whether Best1-mediated glutamate can activate synaptically localized neuronal NMDAR, we recorded mEPSCs from CA1 pyramidal neuron at −60 mV in the presence of tetrodotoxin (TTX) and Bicuculline to isolate miniature EPSCs. We divided mEPSCs into two groups by rise time. Fast rising mEPSCs with 1 ~ 5 ms rise time could be clearly distinguished from slowly rising mEPSCs with 5 ~ 10 ms rise time (Figure 1A, B). The activation of AMPA receptors at synapse occurs on a sub-millisecond time scale, whereas the decay of mEPSCs is much slower, with time constants typically being 10’s of milliseconds. When these mEPSCs occur at significant electrotonic-distance from the recording electrode, the rise of mEPSCs can be distorted or filtered by the dendritic compartment, which acts as a series of RC filters. The consequence of filtering is the slowing of kinetics [29-31]. Therefore, the mEPSCs from more distant places display slower rise (Figure 1C). Another important feature of distal regions of the neuron is that the voltage control can be incomplete, under voltage clamp.

### Astrocytic glutamate does not produce mEPSCs

**Figure 2:**
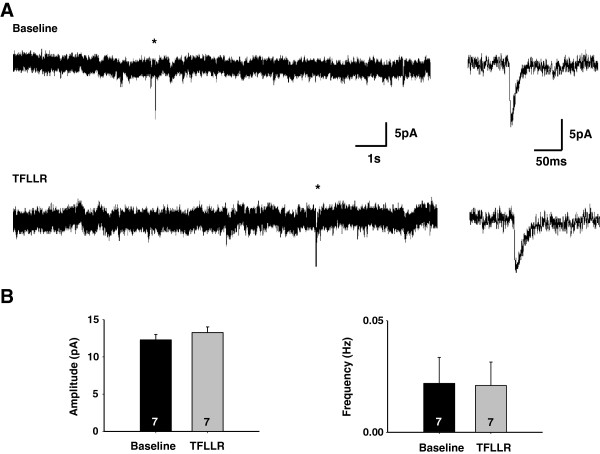
**PAR1 activation does not generate mEPSCs. A**. Hippocampal slices were pretreated with concanamycin A (2 μM ; ~2 hr) to minimize presynaptic glutamate release. The representative traces indicate mEPSCs from the concanmycin A pretreated slices before TFLLR treatment and after treatment, respectively. Asterisk shows mEPSCs. Right : expanded mEPSC traces from basal or TFLLR-treated slices. **B**. Bar graphs represent the averaged amplitudes and frequencies of mEPSCs. Numbers of tested slices from at least two independent mice are indicated in the bar graph. P-value : 0.216 (amplitude), 0.853 (frequency).

PAR1-induced astrocytic glutamate release occurs with a slow time course, on the order of seconds [12]. Therefore, it is unlikely to cause a current that has the appearance of an mEPSC with millisecond kinetics, even though Best1 is expressed at the microdomains of astrocyte near the post-synaptic membrane. To test the possibility that glutamate release from astrocytes can itself generate mEPSC, we recorded under voltage clamp after treating the slices with Concanamycin A (2 μM, at least 2 hrs) to minimize the contribution of neuronal glutamate release, making any potential PAR1-induced mEPSC more readily apparent. To induce astrocytic glutamate release, we used 30 μM TFLLR application to activate PAR1, which is known to be expressed mostly in CA1 hippocampal astrocytes [4,32]. We found that Concanamycin A treatment reduced but did not completely eliminate mEPSCs (Figure 2A, B). This is consistent with the previous report that Concanamycin A treatment without neuronal activity was not able to completely deplete glutamate from presynaptic vesicles from hippocampal Schaffer-collateral presynaptic regions [33]. Under this condition, the induction of glutamate release from astrocytes by TFLLR did not significantly alter the frequency or the peak amplitude of mEPSCs, indicating that astrocytic glutamate release does not generate mEPSCs (Figure 2B).

### Best1-mediated astrocytic glutamate did not affect fast rising mEPSCs

We previously demonstrated that TFLLR-induced astrocytic glutamate affects mEPSC decay kinetics by revealing an APV-sensitive component of the decay in slowly rising miniature EPSC (mEPSC) recorded from CA1 pyramidal neuron, secondary to postsynaptic depolarization and relief of Mg^2+^ block at poorly clamped distal synapses [24]. To test whether Best1 channels expressed in astrocytes are responsible for inducing this NMDAR component of decay in mEPSC, we used a molecular genetic strategy utilizing the Cre-loxP conditional gene silencing system to selectively silence the target gene in the desired cell type [22,34]. To isolate the effect of astrocytic Best1, we used hGFAP-CreERT2 mice regulated by tamoxifen and lentiviral loxP-floxed Best1 shRNA. In this mouse, the CreERT2 is expressed under the control of the GFAP promoter [35]. Best1 shRNA is expressed both in neurons and astrocytes in the absence of tamoxifen, whereas, Best1 shRNA cassette is excised by Cre expression in astrocytes in the presence of tamoxifen, sparing the Best1 in astrocytes. To isolate the effect of Best1 expression in CA1 neurons, we used Ca^2+^/calmodulin-dependent kinase IIα promoter-driven Cre expressing (CaMKIIα-Cre) mice [36] and lentiviral loxP-floxed Best1 shRNA. Under this condition, Best1 shRNA is expressed in all cells, except in CA1 pyramidal neurons. Using cell-type specific Best1 gene silencing system we recorded mEPSCs in CA1 pyramidal neuron.

**Figure 3:**
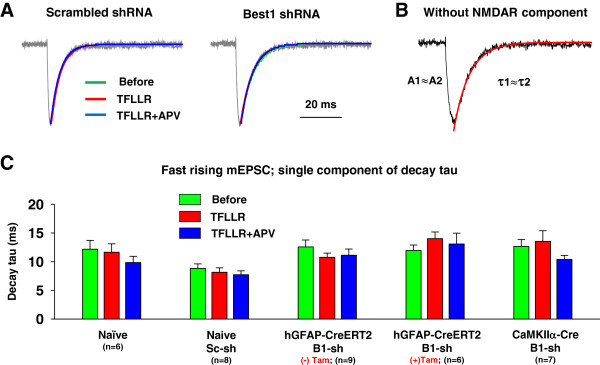
**Decay kinetics of fast rising mEPSCs was not changed by PAR1 activation. A**. Representative superimposed, normalized, averaged mEPSC traces (gray) from fast rising mEPSCs in scrambled shRNA or Best1 shRNA infected slices. The average traces from basal condition (Before; green), TFLLR treated group (TFLLR; red), and TFLLR + APV-treated group (APV; blue) were best fitted with a single component exponential function. **B**. Example of decay fitting by a single exponential function. Fast rising mEPSCs from TFLLR treated hippocampal slices was fitted well by a single component exponential function. Red line indicates decay fitting by a single exponential decay function. NMDAR component is not detected in fast rising mEPSCs. **C**. Bar graphs represent single component of decay tau of fast rising mEPSCs before TFLLR (green), after TFLLR (red), and after TFLLR + APV (blue) treatment in naïve, scrambled shRNA injected naïve, Best1 shRNA injected hGFAP-CreERT2, or Best1 shRNA injected CaMKIIα-Cre mice, respectively. Sc, scrambled shRNA. B1, Best1 shRNA. Tam, tamoxifen. Numbers of tested slices from at least three independent mice are indicated.

**Figure 4:**
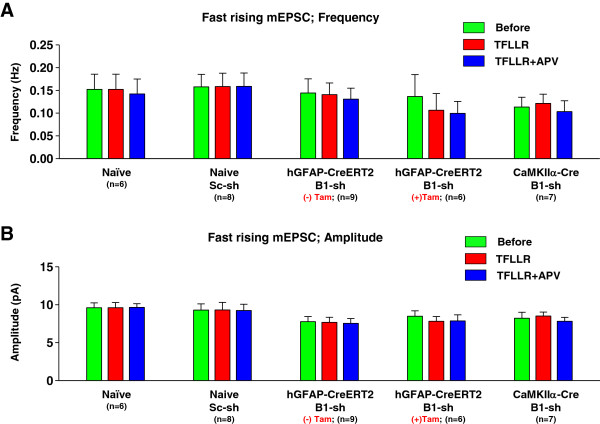
**Frequency and amplitude of fast rising mEPSCs were not changed by PAR1 activation. A, B**) Summary of frequency (**A**) and amplitude (**B**) of fast rising mEPSCs in naïve, scrambled shRNA injected naïve, Best1 shRNA injected hGFAP-CreERT2, or Best1 shRNA injected CaMKIIα-Cre mice, respectively. Both frequency and amplitude were not significantly changed by TFLLR treatment. N indicates the number of tested slices at least three different mice.

Fast rising mEPSCs with a rise time of 1 ~ 5 ms were scaled, averaged, and fitted with a sum of two exponential functions. If the difference between the first decay constant (tau 1) and the second decay constant (tau 2) was less than 10% of the average tau 2 value, the curve was subsequently refitted with a single exponential function [24] (Figure 3A, B). In this case, tau 1 and tau 2 of fast rising mEPSCs by two exponential functions are almost same, because activation of NMDARs is not detected in fast rising mEPSCs occurring in proximal synapse with adequate voltage control (Figure 3B). To assess fit quality, we analyzed traces with the coefficient of determination, R^2^ value above 0.9. If R^2^ value was less than 0.9, we did not include in analysis. The difference between tau 1 and tau 2 of fast rising mEPSCs was mostly within 10%. Therefore, we refitted those with a single exponential function (Figure 3A) and measured decay tau (Figure 3C). The decay tau of fast rising mEPSCs was not significantly changed by TFLLR-induced astrocytic glutamate, suggesting that activation of synaptic NMDARs by Best1-mediated astrocytic glutamate did not appear in fast rising mEPSCs (Figure 3C). Frequency and peak amplitude of fast rising mEPSCs were not affected by Best1-mediated astrocytic glutamate (Figure 4A, B).

### Best1-mediated astrocytic glutamate causes an appearance NMDAR component in slowly rising mEPSCs without affecting frequency and amplitude

**Figure 5:**
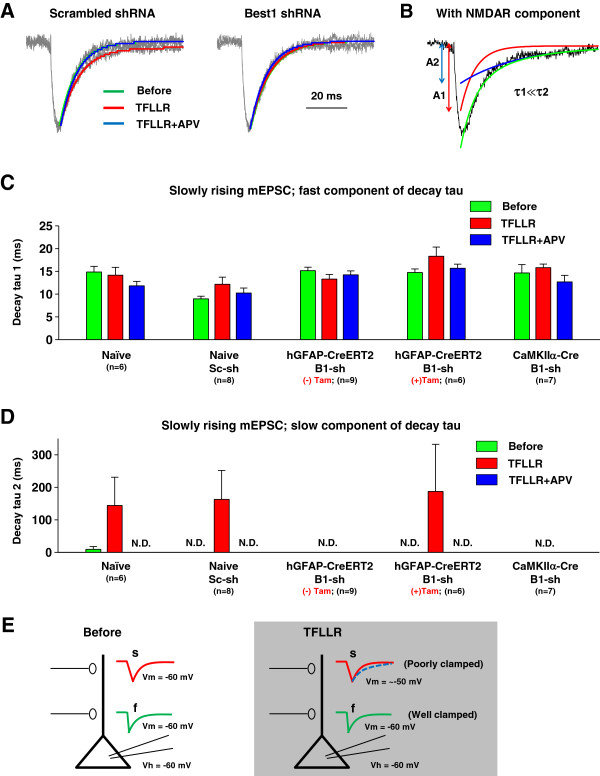
**An appearance of NMDAR component in slowly rising mEPSCs by PAR1 activation. A**. Representative superimposed, normalized, averaged mEPSC traces (gray) from slowly rising mEPSCs in scrambled shRNA or Best1 shRNA infected slices. The average traces from basal condition (left, Before; green) and TFLLR + APV treated group (APV; blue) were best fitted with a single component exponential function. But mEPSC traces from TFLLR-treated group (TFLLR; red) were best fitted with a two component exponential function, which is impaired by Best1 shRNA expression (right). **B**. Example of decay fitting by two component exponential functions in TFLLR treated slices. Red line is decay fitting by single exponential function, and blue line is decay fitting by second exponential decay function. NMDAR component is detected in slowly rising mEPSCs generated in distal dendrite. **C**. Bar graphs represent fast component of decay tau of slowly rising mEPSCs. Tau 1 was not significantly changed by Best1-mediated astrocytic glutamate. **D**. Summary of slow component of decay tau of slowly rising mEPSCs. N.D. indicates not detected by a two exponential function fitting. **E**. Schematic diagram of fast rising and slowly rising mEPSCs in the presence of TFLLR. NMDAR component is not detected in fast rising mEPSCs generating at the proximal dendrites clamped well, whereas activation of NMDAR by TFLLR can be detected at the distal dendrites because of poor voltage clamp.

**Figure 6:**
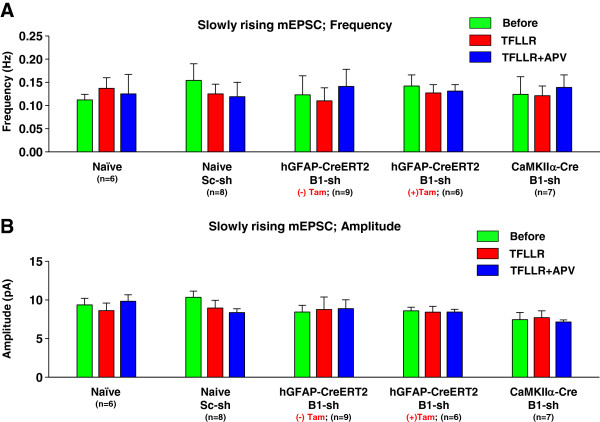
**Frequency and amplitude of slowly rising mEPSCs were not changed by PAR1 activation. A, B**) Summary of frequency (**A**) and amplitude (**B**) of fast rising mEPSCs in respective mice. Both frequency and amplitude were not significantly changed by PAR1 activation.

Next, slowly rising mEPSCs with a rise time of 5 ~ 10 ms were scaled, averaged, and fitted with a sum of two exponential functions (Figure 5A). The difference between tau 1 and tau 2 of slowly rising mEPSCs was more than 10%. We measured both fast component of decay tau (tau 1) and slow component of decay tau (tau 2) of slowly rising mEPSCs (Figure 5B). Fast component of decay tau (tau 1) was not changed by Best1-mediated astrocytic glutamate (Figure 5C). We found that in both naïve and scrambled-shRNA slices, PAR-1 activation elicited an appearance of the NMDAR component, measured as the decay time constant (tau 2) of 2-component exponential decay function model (Figure 5D). The appearance of tau 2 was no longer observed in Best1-shRNA expressing slices from hGFAP-CreERT2 mice without tamoxifen treatment or CaMKIIα-Cre mice (Figure 5D). The disappearance of tau 2 by Best1 gene silencing was mostly rescued in Best1-shRNA-expressing hGFAP-CreERT2 mice treated with tamoxifen (Figure 5D). These results suggest that Best1-mediated glutamate from astrocyte targets synaptic NMDAR located in distal synapses which display slowly rising mEPSCs because of poor voltage control (Figure 5E). Best1-mediated glutamate release from astrocyte did not affect either the frequency or peak amplitude of slowly rising mEPSCs (Figure 6A, B). These results indicate that Best1 channels expressed in astrocytes are responsible for the appearance of NMDAR component in decay of mEPSCs, and indicate that Best1-mediated glutamate release from astrocytes can target synaptically localized NMDARs.

## Discussion

The important finding of this study is that astrocytically released glutamate via Best1 targets and activates synaptically localized NMDARs in hippocampal formation. In addition, we demonstrated that the appearance of NMDAR component in the decay of AMPAR-mediated mEPSC occurred in a specific population of mEPSCs that originated from distal dendrites rather than proximal dendrites; these mEPSCs slowly rose due to the dendritic RC filtering, which we used as a marker for distal mEPSCs.

The distance from the recording electrode also affects the ability to voltage clamp under voltage clamp configuration by the virtue of space clamp problem. The distal synapses can be less well clamped and can easily escape from voltage clamping under depolarizing stimulus, whereas proximal synapses can be better voltage clamped. When there is a depolarizing condition such as astrocytic glutamate release that leads to depolarization of distal synapses due to extrasynaptic NMDA receptor activation, membrane voltage can escape from voltage clamp and allow the synaptic NMDA receptors to relieve the Mg^2+^ block. This phenomenon becomes more pronounced when there is a co-incidence of presynaptic quantal release to activate synaptic AMPA receptors to cause a depolarization to instantly relieve the Mg^2+^ block of NMDA receptors during a mEPSC. Therefore, due to space clamp problem, the mEPSCs occurring at distal synapses show pronounced NMDA receptor component of the mEPSCs as evidenced by the appearance of 2^nd^ exponential decay component in the decay phase of mEPSCs.

In our previous paper, we have demonstrated that TFLLR activation of PAR1 causes astrocytic glutamate release and this release can be detected as an appearance of the NMDAR component in the decay of mEPSCs, as evidenced by the presence of 2^nd^ exponential function in the decay fitting [24]. The appearance of APV-sensitive NMDAR mediated slow component that rides on top of the decay kinetics of AMPA receptor component of mEPSCs is induced by TFLLR application only in mEPSCs from distal dendrites with slow rise, but not from fast rise mEPSCs that come from proximal dendrites. Synaptic NMDAR acts as a coincidence detector when there is a coincidental depolarization by synaptic AMPAR and this feature originates from the ability to relieve the Mg^2+^ block. This is due to the incomplete voltage clamp in distal dendrites that allows voltage escape and subsequent relief from the Mg^2+^ block of synaptic NMDA receptors.

All mEPSCs, slowly rising or fast rising mEPSCs, originate from neuronal glutamate release from the terminals. The frequency of mEPSCs allows us to directly monitor what goes on at individual synapse, which contains both AMPAR and NMDAR. Under normal condition both slowly rising and fast rising mEPSCs recorded under voltage clamp only display AMPAR mediated component, with no apparent NMDAR component due to strong Mg^2+^ block at holding potential of −60 mV or so (Figure 5D, E). When there is TFLLR-induced glutamate release from astrocytes close to or at the synapse, the astrocytic glutamate does not produce an “mEPSC-like” waveform, but instead diffuses slowly at low concentration (1 uM), binds to and activating NMDAR slowly. We propose that continued activation at low level of NMDA receptors (likely receptors with reduced Mg^2+^ sensitivity, including NR2D or NR3 subunits) can produce a small inward current that will depolarize distal dendritic spines. Normally NMDAR are under strong Mg^2+^ block. However, with the tonic low level of NMDA receptor activation following astrocytic release of glutamate, there will be a reduction on voltage dependent Mg^2+^ block such that release of glutamate from presynaptic terminal, will activate the synaptic NMDARs and this opening shows up in the decay phase of mEPSC. This is only possible when voltage can escape from the holding voltage during the synaptic release of glutamate. This voltage escape is pronounced (and detectable) in the distal synapses where voltage clamping is hard to achieve. Therefore, the fast rising (well-clamped) mEPSCs do not show this NMDAR component by TFLLR, whereas the slowly rising (poorly clamped) distal synapses do show the NMDA receptor component.

It is important to note that even though the fast rising mEPSCs do not show NMDAR component upon TFLLR application, this does not necessarily mean that astrocytic glutamate release via Best1 channels is absent in the proximally located synapses. The reason the NMDAR component is not prominent in fast rising mEPSCs is because the proximal dendrites are well-clamped under voltage clamp configuration. However, under the natural condition where membrane voltage is free to change, it is expected that distal as well as proximal synaptic events are equally affected by astrocytic glutamate release via Best1 channel. Therefore, astrocytic glutamate release should globally affect the synaptic NMDAR, regardless of the location of synapses along a dendrite.

Best1-mediated glutamate from astrocyte targets NMDARs not AMPARs. In our recent study, we calculated the concentration of glutamate released by astrocyte via Best1 channel. The concentration of Best1 mediated glutamate release is around 1 μM [12]. This concentration is just enough to activate NMDA receptors (EC50: ~ 3 μM) but not sufficient to activate AMPA receptors (EC50: ~ 1000 μM). In addition, AMPA receptors are highly desensitizing, and the decay kinetics of mEPSCs is strongly influenced by the desensitizing kinetics of the AMPA receptors. Nevertheless, there is a possibility that 1 μM glutamate can activate synaptic AMPA receptors, although the effect may not be apparent due to strong desensitization.

It is also worthwhile to note that whether the glutamate released by Best1 channel binds directly to synaptically localized NMDAR or not is still not clear. Considering the fact that Best1 channel is specifically localized at the microdomains of astrocytes near synaptic junctions [12], one can predict that glutamate released by Best1 channel should directly activate the synaptically localized NMDARs at the post synapse. On the other hand, it is equally possible that astrocytic glutamate can activate extra-synaptic NMDARs and then subsequently affect the synaptic NMDARs. In this study, we could not distinguish the two possibilities. For future studies, it will be interesting to test the effect of activity-dependent pore block of synaptic NMDARs using the MK-801 to distinguish the two possibilites.

In summary, we demonstrated that the TFLLR-induced appearance of NMDAR-mediated, slow component of mEPSC in the subpopulation of slowly rising mEPSCs is due to astrocytic glutamate release through Best1 channels that specifically target the synaptically localized NMDAR.

## Methods

### Delivery of lentiviral vector containing Best1 shRNA into mouse hippocampus

hGFAP-CreERT2 transgenic mice was provided by Dr. Ken McCarthy. CaMKIIα-Cre transgenic mice were purchased from Jackson Laboratory. hGFAP-CreERT2 mice were used at the age of 7 weeks for tamoxifen or sun flower oil injection (intraperitoneal injection, once per day for 5 days). Then the lentivirus carrying Best1 shRNA was injected 1 day after 5^th^ day injection. Finally the mice were sacrificed for mEPSC recordings at 7 ~ 9 weeks of age. CaMKIIα-Cre was used at 7 ~ 8 weeks of age for virus injection and used at around 9 weeks for electrophysiological recordings. Only male mice were used in this study.

### Slice preparation

Adult mice (7 ~ 9 weeks) were deeply anaesthetized until cessation of breathing and subsequently decapitated. The brain was rapidly removed and submerged in an ice-cold oxygenated artificial cerebrospinal fluid (ACSF) composed of (in mM) 130 NaCl, 24 NaHCO_3_, 3.5 KCl, 1.25 NaH_2_PO_4_, 1 CaCl_2_, 3 MgCl_2,_ 10 glucose at pH 7.4, and was bubbled with 5% CO_2_ / 95% O_2_. Transverse mouse brain slices (350 ~ 400 μm) containing hippocampus were acutely prepared with a Leica vibratome (Leica VT1000S), and incubated in a chamber with oxygenated ACSF at room temperature for 1 hr before use.

### Recording of mEPSCs

The standard ACSF recording solution was composed of (mM): 130 NaCl, 24 NaHCO_3_, 3.5 KCl, 1.25 NaH_2_PO_4_, 1.5 CaCl_2_, 1.5 MgCl_2_ and 10 glucose saturated with 95% O_2_–5% CO_2_, at pH 7.4. The internal solution was composed of (mM): 140 Cs-MeSO_4_, 10 Hepes, 7 NaCl, 4 Mg-ATP and 0.3 Na_3_-GTP. Visually guided whole-cell patch recordings were obtained from CA1 pyramidal neurons.

Recordings were obtained using Axopatch 200A (Axon instruments, Union City, CA, USA) and filtered at 2 kHz. In case of mEPSC recording, recordings were digitized at 10 kHz, and analyzed using pCLAMP 9 (Molecular devices) and Mini Analysis Program (Synaptosoft) as previously described [24]. The mEPSCs were automatically detected and grouped as fast (1–5 ms) and slow rise time (5–10 ms). All experimental procedures described were performed in accordance with the institutional guidelines of Korea Institute of Science and Technology (KIST, Seoul, Korea).

## Competing interests

The authors declare that they have no competing interests.

## Authors’ contributions

KSH performed and analyzed the most of experiments and wrote the manuscript. JW measured mEPSC in the presence of concanamycin A. HP analyzed mEPSCs data. BJY and SC analyzed the data. CJL and SC designed the most of experiments and wrote the manuscript, and supervised entire project. All authors read and approved the final manuscript.
